# Integrated analysis of the molecular mechanisms in idiopathic pulmonary fibrosis

**DOI:** 10.7150/ijms.61309

**Published:** 2021-08-02

**Authors:** Ke Zhu, Aiqun Xu, Wanli Xia, Pulin Li, Rui Han, Enze Wang, Sijing Zhou, Ran Wang

**Affiliations:** 1 Department of Respiratory and Critical Care Medicine, The First Affiliated Hospital of Anhui Medical University, Hefei 230022, China; 2 Department of General Medicine, Hefei Second People's Hospital, Hefei 230001, China; 3 Department of Thoracic Surgery, the first affiliated hospital of Anhui medical university, Hefei 230022, China; 4 Hefei Third Clinical College of Anhui Medical University, Hefei 230022, China.; 5 Hefei Prevention and Treatment Center for Occupational Diseases, Hefei 230022, China.

**Keywords:** IPF, Lung tissue, Genes, microRNAs, Bioinformatics

## Abstract

**Rationale:** Idiopathic pulmonary fibrosis (IPF) is one of the most aggressive forms of idiopathic interstitial pneumonia. Some miRNAs may be associated with IPF and may affect the occurrence and development of IPF in various pathways. Many miRNAs and genes that may be involved in the development of IPF have been discovered using chip and high throughput technologies.

**Methods:** We analyzed one miRNA and four mRNA databases. We identified hub genes and pathways related to IPF using GO, KEGG enrichment analysis, gene set variation analysis (GSVA), PPI network construction, and hub gene analysis. A comprehensive analysis of differentially expressed miRNAs (DEMs), predicted miRNA target genes, and differentially expressed genes (DEGs) led to the creation of a miRNA-mRNA regulatory network in IPF.

**Results:** We found 203 DEGs and 165 DEMs that were associated with IPF. The findings of enrichment analyses showed that these DEGs were mainly involved in antimicrobial humoral response, antimicrobial humoral immune response mediated by antimicrobial peptide, extracellular matrix organization, cell killing, and organ or tissue specific immune response. The VEGFA, CDH5, and WNT3A genes overlapped between hub genes and the miRNA-mRNA regulatory network. The miRNAs including miR-199b-5p, miR-140-5p, miR-199a-5p, miR-125A-5p, and miR-107 that we predicted would regulate the VEGFA, CDH5, and WNT3A genes, which were also associated with IPF or other fibrosis-related diseases. GSVA indicated that metabolic processes of UTP and IMP, immune response, regulation of Th2 cell cytokine production, and positive regulation of NK cell-mediated immunity are associated with the pathogenesis and treatment of IPF. These pathways also interact with VEGFA, CDH5, and WNT3A.

**Conclusion:** These findings provide a new research direction for the diagnosis and treatment of IPF.

## Introduction

The four types of idiopathic interstitial pneumonia (IIP) comprise a nonspecific type with fibrosis, and the usual, acute, and desquamative types 1. Idiopathic pulmonary fibrosis (IPF) is one of the most aggressive forms of IIP 2. The etiology of IPF is unknown and it mainly occurs in elderly persons 3. The disease course of IPF is variable and unpredictable, but progression to end-stage respiratory insufficiency and death usually occurs 2-4 years after the onset of symptoms and diagnosis 4. The lives of patients with IPF who experience acute respiratory deterioration are affected 5. Idiopathic interstitial pneumonia has attracted considerably more attention than other forms of lung interstitial disease due to its poor prognosis and lack of response to conventional therapies 6. Therefore, the mechanisms of IPF should be clarified and relevant biomarkers should be identified to increase diagnostic accuracy and develop more effective treatments for IPF.

MicroRNAs (miRNAs or miRs) generally comprise 18-23 nucleotides, and they regulate gene activity at the post-transcriptional and translational levels 7. Some miRNAs may regulate the occurrence and development of IPF by regulating fibroblast proliferation, collagen production, and other pathways 8, 9.

Complex networks of molecular processes lead to diseases such as IPF. Gene chips (DNA microarrays) have become important for rapidly acquiring large-scale information on gene expression profiles 10. Many genes that might be associated with the development of IPF have recently been discovered using chip and high throughput technologies 11. However, the results of various studies are limited or inconsistent, due to differences in technology platforms or insufficient sample size. Nonetheless, large amounts of valuable biological information can be generated via integrative analysis using bioinformatic methods 12. The results of a series of analyses may provide insight into the diagnosis and treatment of IPF. Therefore, the present study aimed to identify differentially expressed genes (DEGs) and differentially expressed miRNAs (DEMs) between normal and IPF lung tissues using mRNA and miRNA expression profiles downloaded from the Gene Expression Omnibus (GEO) 13 and a bioinformatic approach.

## Methods

### Microarray data

We included datasets to compare mRNA or miRNA expression in IPF and normal tissues. Search terms approximately comprised “idiopathic pulmonary fibrosis” and “idiopathic interstitial pneumonia”. We first read the title and summary of the datasets, selected those of interest, and then further evaluated all the information in the datasets to select the most appropriate datasets.

The inclusion criteria included:

(1) The source of each dataset is the microarray data of total RNA or miRNA extracted from the lung tissue of IPF patients;

(2) Samples were obtained in the same way;

(3) The dataset should contain two sets of data, namely the IPF group and the control group;

(4) The number of samples in the dataset should be greater than or equal to 10;

(5) The dataset should provide raw data or matrix file that can be processed to obtain the appropriate LogFC values;

(6) The quality of the dataset should be qualified to obtain enough DEGs, and the DEGs can effectively divide the samples into IPF group and control group.

The exclusion criteria included:

(1) The samples of the dataset are not lung tissue;

(2) The data of the dataset is not microarray data;

(3) No control group;

(4) Experiments on animals;

(5) The number of samples is less than 10.

Finally, the miRNA dataset GSE21394 14 and the mRNA datasets GSE10667 15, GSE15197 16, GSE47460 17, and GSE110147 18 were selected from the GEO database (https://www.ncbi.nlm.nih.gov/geo) for further investigation. GSE21394 was based on GPL8936 (Agilent-019118 Human miRNA Microarray 2.0 G4470B (Probe Name version)) platform. GSE10667 was based on GPL4133 (Agilent-014850 Whole Human Genome Microarray 4x44K G4112F (Feature Number version). GSE15197 and GSE47460 were based on the GPL6480 (Agilent-014850 Whole Human Genome Microarray 4x44K G4112F; Probe Name version), and GSE110147 was based on GPL6244 ([HuGene-1_0-st] Affymetrix Human Gene 1.0 ST Array transcript [gene] version) platform. We obtained the following IPF and control tissues as follows, respectively: nine and five from GSE21394, 23 and 14 from GSE10667, eight and 12 from GSE15197, 33 and 17 from GSE47460, and 22 and 11 from GSE110147.

### Identification of DEGs

We corrected the backgrounds, standardized and normalized GSE10667, GSE15197, and GSE47460 using the limma package (Version: 3.48.0) in R (http://www.bioconductor.org/packages/release/bioc/html/limma.html), and the affyPLM package (Version: 1.68.0) in R (http://www.bioconductor.org/packages/release/bioc/html/affyPLM.html) for GSE110147 and created a probe gene expression matrix. Among them, “normexp” 19 and “quantile” 20 were used for background correction and normalization of Agilent datasets, while Robust Multichip Average (RMA) 21 was used for the processing of GSE110147. We converted probes into corresponding genetic symbols using Perl (https://www.perl.org/). We used perl script to convert the probe IDs in the probe expression matrices to the corresponding gene symbols in the platform files 22. Then, we used Limma package to analyze each obtained matrix file of gene symbols, calculated p values and |logFC|, and screen differential genes according to same criteria 23. Our criteria for screening DEGs were P < 0.05 and an absolute value of logFC > 1. The RobustRankAggreg (RRA) package (Version: 1.1) in R (https://cran.rstudio.com/bin/windows/contrib/4.1/RobustRankAggreg_1.1.zip) reviews the sequences of each gene in each list and assumes that each gene identified in each experiment is randomly arranged; this is suitable for comparing multiple sequencing gene lists 24. We used the RRA package to integrate the four TXT files of all genes in logFC sequencing, and saved the integrated upregulated and downregulated DEG lists for subsequent analysis.

### Gene Ontology (GO) and Kyoto Encyclopedia of Genes and Genomes (KEGG) enrichment analyses of DEGs

We used R software to analyze the pathways of GO and KEGG. The “org.Hs.eg.db” package (Version: 3.13.0; http://www.bioconductor.org/packages/release/data/annotation/html/org.Hs.eg.db.html) was used to convert the gene symbols to entrezIDs 25. Packages used for GO and KEGG enrichment analyses include “clusterProfiler” (Version: 4.0.0; http://www.bioconductor.org/packages/release/bioc/html/clusterProfiler.html), “ggplot2” (Version: 3.3.3; https://cran.r-project.org/web/packages/ggplot2/) and “enrichplot” (Version: 1.12.0; http://www.bioconductor.org/packages/release/bioc/html/enrichplot.html) 26. The utility database KEGG is a resource for understanding advanced functional and biological systems (such as cells, organisms, and ecosystems) 27. This database also comprises a large dataset of information generated at the molecular level, especially by genome sequencing and other high-throughput experimental technologies 27. GO is popular in bioinformatics, as it covers the following aspects of biology: cellular components (CCs), molecular functions (MFs), and biological processes (BPs) 28. Values with P < 0.05 were considered statistically significant.

### Gene Set Variation Analysis (GSVA)

GSVA is a gene set enrichment method that estimates variations in pathway activity over a sample population without supervision 29. We downloaded the GO and KEGG databases of gene sets from the Molecular Signatures Database on the GSEA website (https://www.gsea-msigdb.org/gsea/index.jsp). During the above screening for DEGs, we obtained a matrix file of gene symbols. Using GSVA (Version: 1.40.0) (http://www.bioconductor.org/packages/release/bioc/html/GSVA.html), limma, and GSEABase (Version: 1.54.0) (http://www.bioconductor.org/packages/release/bioc/html/GSEABase.html) in R, we processed the matrix files of gene symbol and GO and KEGG databases and the pathways and functions were scored according to the degree of absolute enrichment of a gene set in each sample 30. After that, we obtained the functions and pathways matrix files of GO and KEGG. The main content of the matrix files was the GSVA score for each function or pathway corresponding to each sample. After obtaining the matrix files of GO and KEGG, we used limma to analyze differential GSVA scores between IPF and normal samples, and then calculated p values and |logFC|. Our criteria for screening the differential GSVA scores were P < 0.05 and an absolute value of logFC > 0.2. We used RRA to integrate the four TXT files of all functions or pathways in logFC sequencing, and saved the upregulated and downregulated differentially expressed functions and pathway lists for subsequent analysis.

### Protein-protein interaction network construction and Hub gene analysis

We used the Search Tool for the Retrieval of Interacting Genes/Proteins (STRING) database (http://string-db.org/) to analyze PPI information 31. The STRING database collects, scores, and integrates all publicly available sources of protein-protein interaction information, and supplements them with computational predictions 32. We mapped known DEGs to the STRING database to determine potential PPI relationships. Combined scores of interactions > 0.4 were considered statistically significant. Cytoscape analyzes and visualizes massive networks and provides greater flexibility in importing additional data into, and visualizing data in the network 33. CytoHubba is a Cytoscape plugin that explores PPI network hub genes 34. We identified the top 20 scoring genes as hub genes.

### Identification of DEMs

We corrected the background, standardized, and normalized GSE21394 using limma R and obtained a probe gene expression matrix. We converted probes into corresponding genetic symbols using Perl. After obtaining the matrix file of gene symbols, we analyzed the differential expression of miRNAs between IPF and normal samples using limma R, and then calculated p values and |logFC|. Our criteria for screening DEMs were P < 0.05 and an absolute value of logFC > 1.

### Prediction of target genes of DEMs and intersection of DEGs

Functional enrichment (FunRich) is an analytical tool for gene or protein functional enrichment and protein-protein interaction network analysis 35. The microRNA enrichment function in FunRich can be used to analyze miRNA enrichment, predict microRNA targets, or identify microRNAs through given target genes 35. Functions of the DEM target genes were analyzed to predict target genes using the FunRich 35 database (http://www.funrich.org/). We obtained a list of miRNAs and predicted target mRNAs. Target gene, DEG, and DEM data were integrated using Perl to acquire information about DEM and DEG interactions.

### Construction of miRNA-mRNA network

Interactions between DEMs and DEGs was analyzed using Cytoscape, and a network between miRNAs and mRNAs was constructed.

## Results

### Identification of DEGs

**Figure 1:**
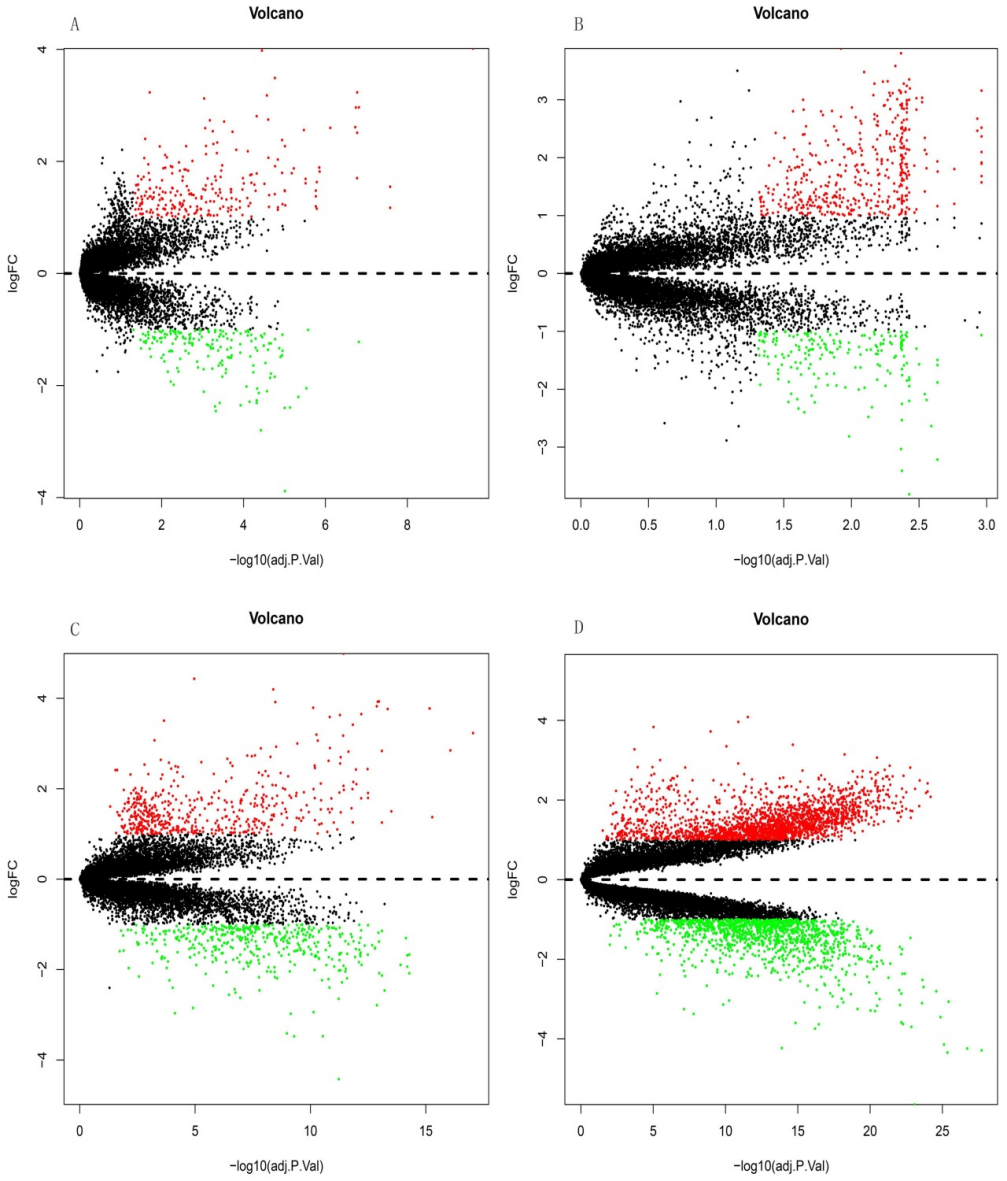
Differential expression analysis of DEGs in GSE10667, GSE15197, GSE47460, and GSE110147 using volcano plots. DEGs in (A) GSE10667 show 239 upregulated and 172 downregulated genes. (B) GSE15197 shows 451 upregulated and 192 downregulated genes. (C) GSE47460 shows 512 upregulated and 427 downregulated genes. (D) GSE110147 shows 2074 upregulated and 1300 downregulated genes. Red, green, and black dots respectively indicate downregulated, upregulated, and not significantly changed mRNAs.

**Figure 2:**
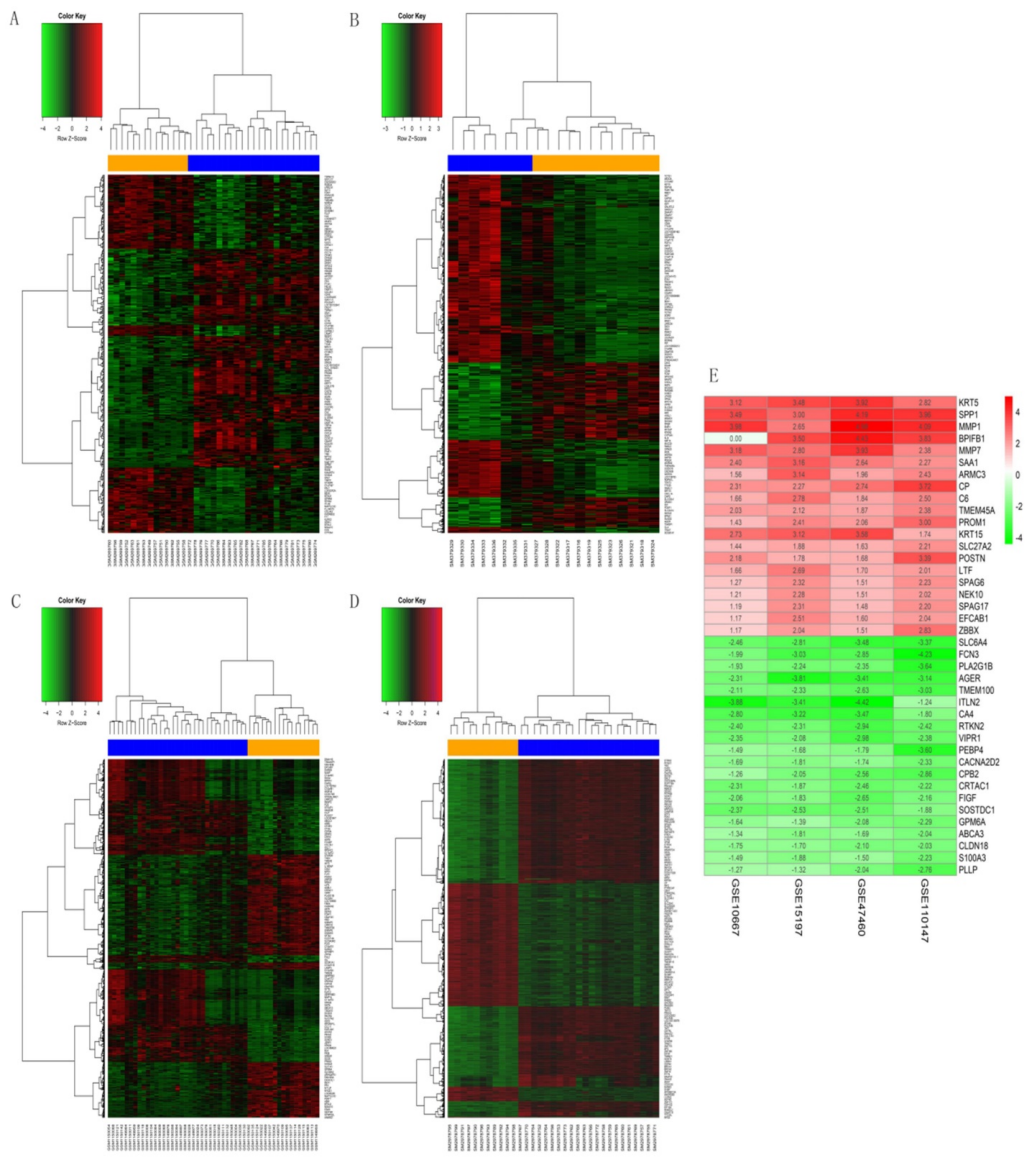
Heatmaps of DEGs in GSE10667, GSE15197, GSE47460, and GSE110147. (A) GSE10667, (B) GSE15197, (C) GSE47460, (D) GSE110147. (E) RobustRankAggreg (RRA) of four mRNA datasets. A-D: Blue, IPF; yellow, normal lung tissues. A-E: red and green, upregulated genes and downregulated genes, respectively.

The GSE10667 database revealed 411 DEGs (239 and 172 upregulated and downregulated, respectively) that were expressed > 2-fold in IPF tissues compared with controls (Figures 1A and 2A). The GSE15197 database showed 643 DEGs (451 and 192 upregulated and downregulated, respectively) that were expressed > 2-fold in IPF tissues compared with controls (Figures 1B and 2B). The GSE47460 database contained 939 DEGs (512 and 427 upregulated and downregulated, respective) that were expressed > 2-fold in IPF tissues compared with controls (Figures 1C and 2C). We used the same method to analyze the GSE110147 database and identified 3,374 DEGs, among which 2,074 and 1300 were upregulated and downregulated, respectively compared with the control group (Figures 1D and 2D). The RRA method also uncovered 203 integrated DEGs, comprising 92 and 111 that were upregulated and downregulated, respectively (Table S1). The first 20 each of upregulated and downregulated genes were plotted on a heatmap (Figure 2E).

### Pathway enrichment findings

**Figure 3:**
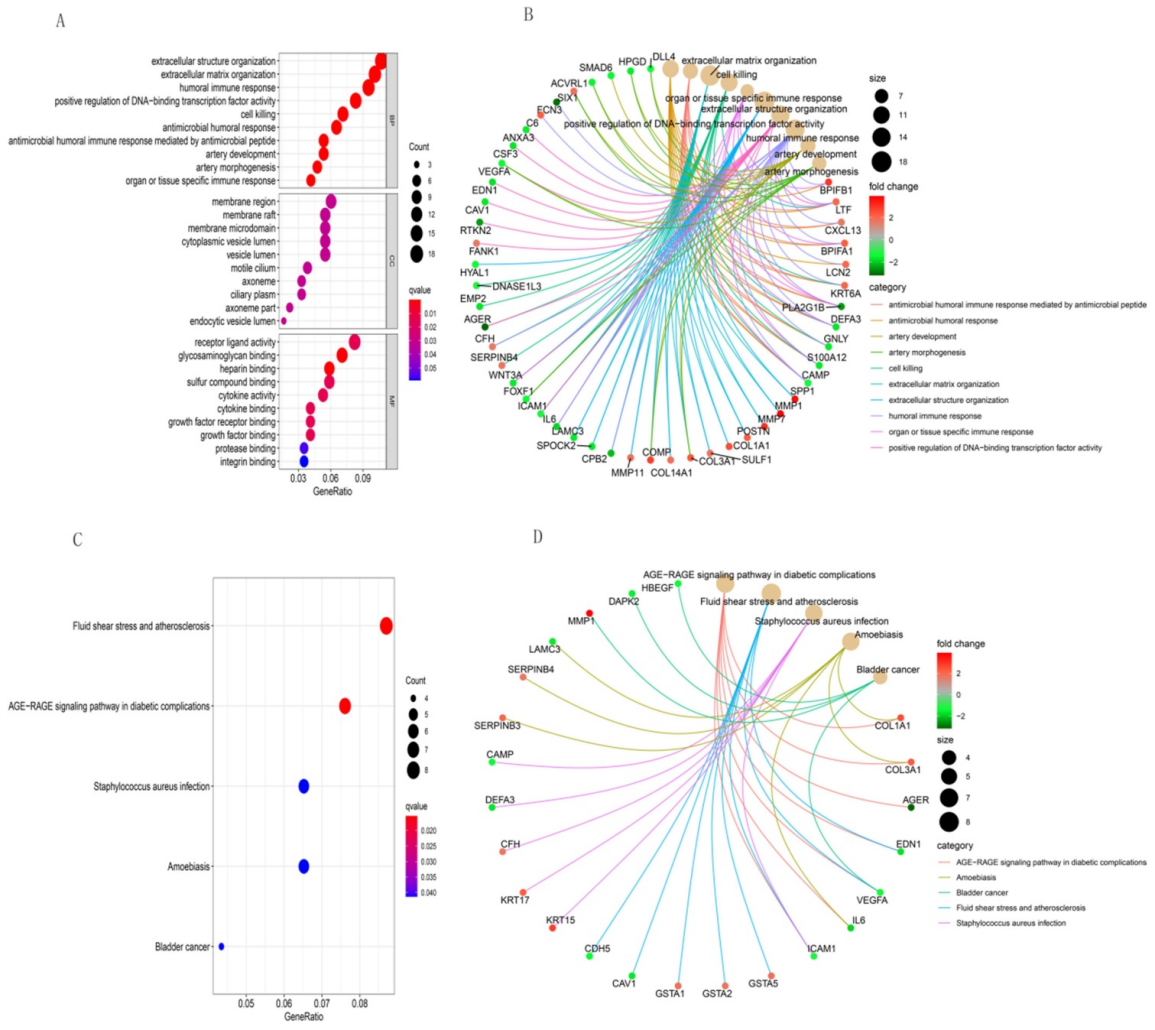
GO and KEGG pathway enrichment analyses of integrative DEGs. GO functional annotation of (A) biological processes, cellular components, and molecular functions of integrative DEGs. (B) The most significant GO functions. (C) and (D) KEGG enrichment analysis of integrative DEG pathways.

We used R software to analyze the functional and pathway enrichment of 203 DEGs and determine their biological activities. Figure 3 shows the findings of BP, CC, MF, and KEGG pathways. Antimicrobial humoral response, antimicrobial humoral immune response mediated by antimicrobial peptide, extracellular matrix organization, cell killing, organ or tissue specific immune response and extracellular structure organization were the most enriched terms in BP (Figure 3A). Those in CC were membrane region, axoneme part, membrane raft, membrane microdomain, and endocytic vesicle lumen (Figure 3A). The most enriched terms in MF were glycosaminoglycan binding, heparin binding, sulfur compound binding, receptor ligand activity, and cytokine binding (Figure 3A). The most enriched GO functions according to the size of the different FDR values were shown in Figure 3B, and those in KEGG were AGE-RAGE signaling pathway in diabetic complications, fluid shear stress and atherosclerosis, staphylococcus aureus infection, amoebiasis, and bladder cancer (Figure 3C and 3D).

### GSVA analysis

**Figure 4:**
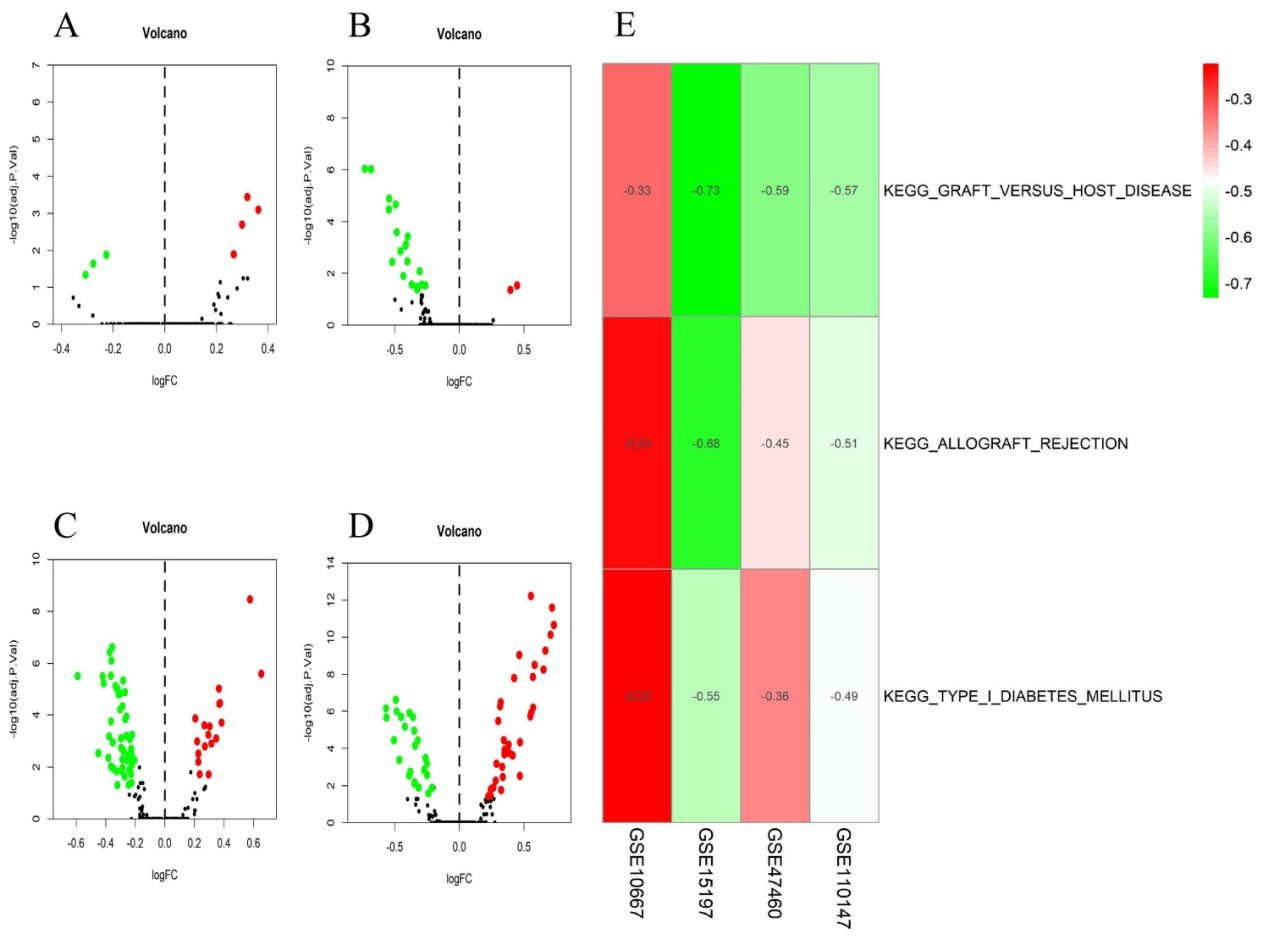
Volcano plots of differential GSVA scores for KEGG terms (A-D) and heatmap of RRA findings (E). (A) GSE10667: four upregulated and three downregulated. (B) GSE15197: two upregulated and 19 downregulated. (C) GSE47460: 18 upregulated and 50 downregulated. (D) GSE110147: 33 upregulated and 24 downregulated. (E) Heatmap of GSVA scores (KEGG) of four datasets using RRA. Red and green: upregulated and downregulated KEGG terms (dots A-D) and RRA findings (E).

**Figure 5:**
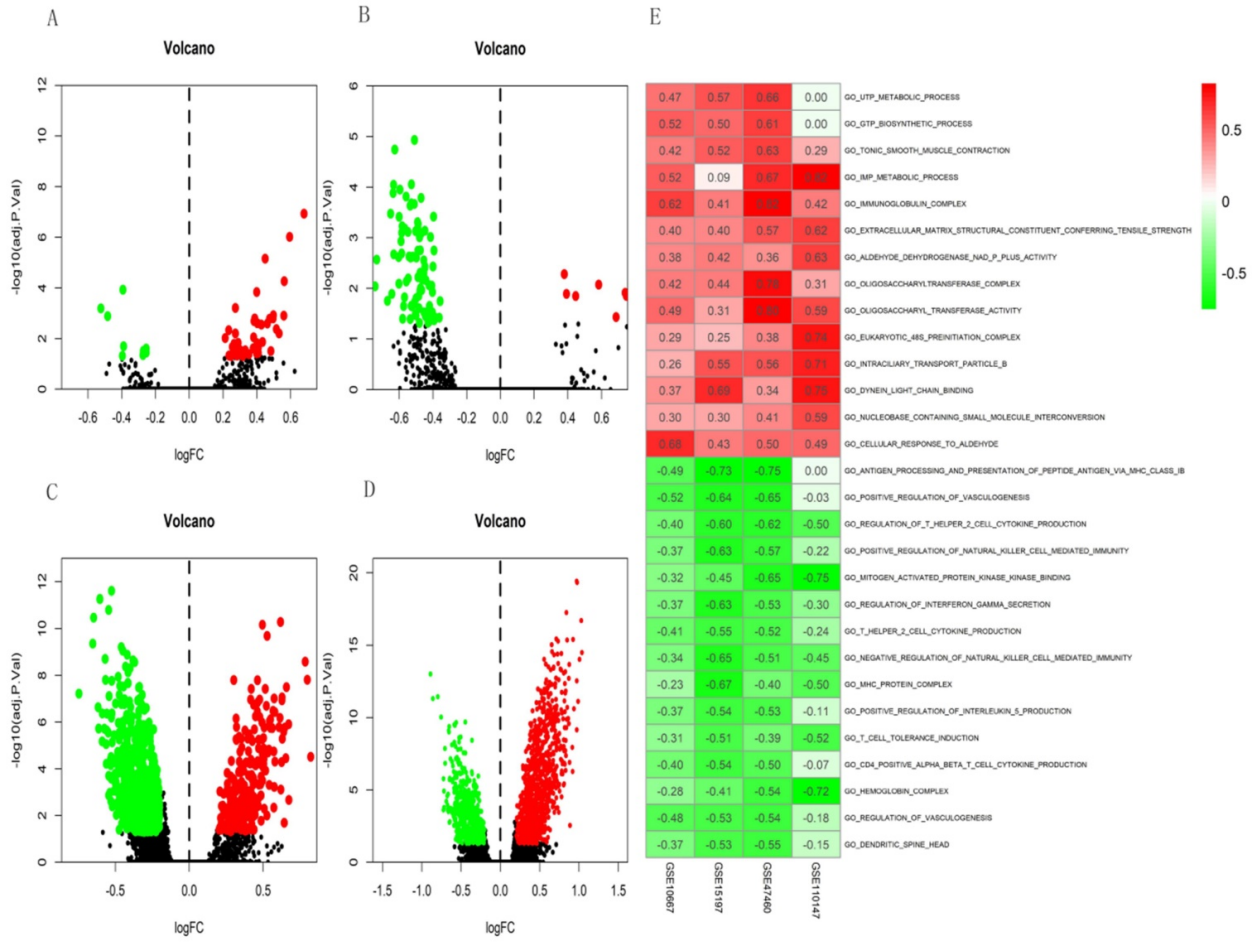
Volcano plots of differential GSVA scores for GO terms (A-D) and heatmap (E) of four datasets. (A) GSE10667: 46 upregulated and nine downregulated. (B) GSE15197: 10 upregulated and 84 downregulated. (C) GSE47460: 257 upregulated and 553 downregulated. (D) GSE110147: 1001 upregulated and 519 downregulated. E. Heatmap of GSVA scores (GO) of four datasets using RRA. In Figure 5E, red represents upregulated GO terms and green represents downregulated GO terms.

The differential scores of seven (four upregulated and 3 downregulated) KEGG (Figure 4A) and 55 (46 upregulated, and nine downregulated) GO terms (Figure 5A) in the GSE10667 database significantly differed between control and IPF tissue samples. We found 21 (two upregulated and 19 downregulated) KEGG terms (Figure 4B) and 94 (10 upregulated, and 84 downregulated) GO terms in the GSE15197 dataset (Figure 5B). We found 68 (18 upregulated, and 50 downregulated) KEGG terms (Figure 4C) and 810 (257 upregulated and 553 downregulated GO) terms in the GSE47460 dataset (Figure 5C). We identified 57 (33 upregulated and 24 downregulated) KEGG terms (Figure 4D) and 1,520 (1001 upregulated and 519 downregulated) GO terms in the GSE110147 dataset (Figure 5D). The RRA findings revealed three KEGG (Tables S2 and 3, Figure 4E) and 29 GO differential functions and pathways (Figure 5E). The three KEGG functions were inhibitory (graft versus host disease, allograft rejection, type I diabetes mellitus), whereas the 14 GO pathways and functions were activated; the functions and pathways with the most significant differences were UTP metabolic process, GTP biosynthetic process, tonic smooth muscle contraction, IMP metabolic process, immunoglobulin complex), and 15 inhibited pathways and functions; the five functions and pathways with the most significant differences were antigen processing and presentation of peptide antigen via MHC class IB, positive regulation of vasculogenesis, regulation of T helper 2 cell cytokine production, positive regulation of natural killer cell-mediated immunity, and MEK binding.

### PPI network construction and Hub gene analysis

**Figure 6:**
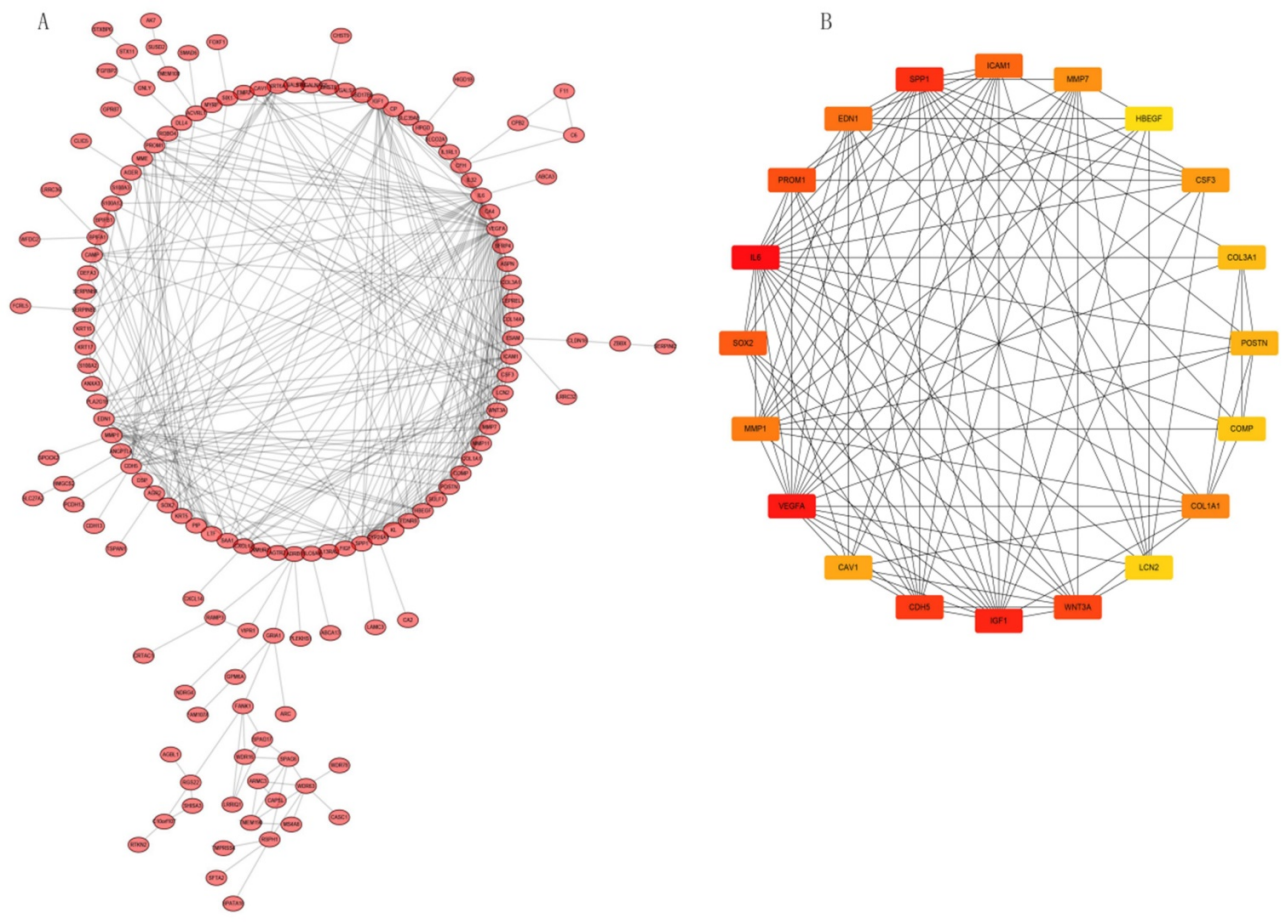
PPI network constructed with integrated DEGs and hub gene analysis. (A) Interaction network of all integrated DEGs. (B) Top 20 hub genes.

We analyzed interactions among 203 DEGs using STRING (version 11.0) to identify physical PPIs between the underlying nodes of IPF. We evaluated the data using Cytoscape (Figure 6A) and analyzed the hub genes of MCC using the cytoHubba plugin. Twenty genes with the highest scores (IL6, VEGFA, IGF1, SPP1, CDH5, WNT3A, PROM1, SOX2, ICAM1, EDN1, MMP1, COL1A1, MMP7, CSF3, CAV1, POSTN, COL3A1, COMP, LCN2, and HBEGF) were identified as hub genes (Figure 6B), suggesting that these genes play roles in the occurrence and development of IPF. The 20 hub genes comprised 11 upregulated (IGF1, SPP1, PROM1, SOX2, MMP1, COL1A1, MMP7, POSTN, COL3A1, COMP, LCN2) and nine downregulated (IL6, VEGFA, CDH5, WNT3A, ICAM1, EDN1, CSF3, CAV1, HBEGF) genes.

### Identification of DEMs

**Figure 7:**
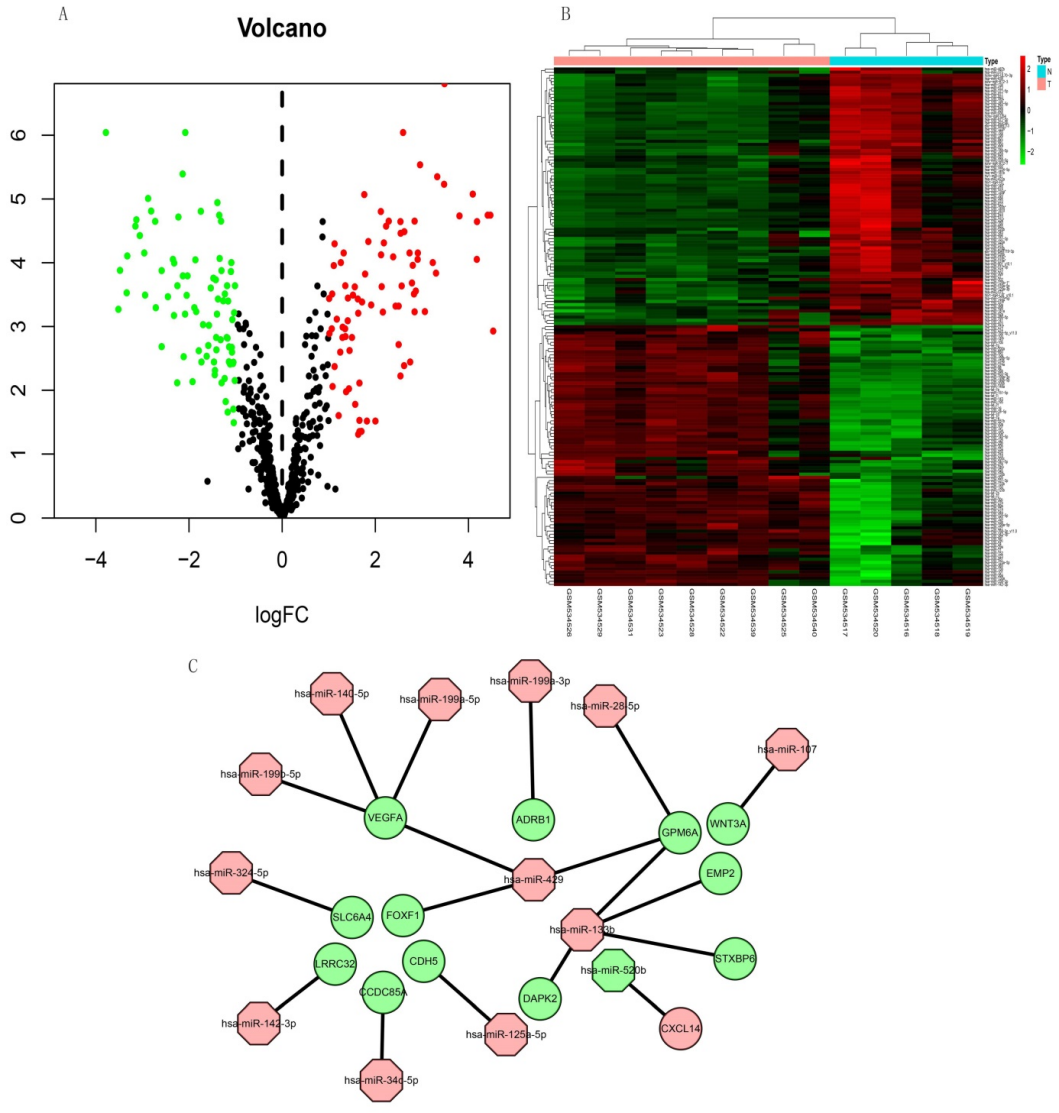
Volcano plot and heat map of DEMs in miRNA dataset GSE21394. (A) Volcano plot shows 83 upregulated (red dots), 82 downregulated (green dots), and not significantly changed mRNAs (black dots). (B) Heatmap of the DE-miRNAs of GSE21394. (C) Regulatory miRNA-mRNA network. Red and green, upregulation and downregulation, respectively. N, normal tissues; T, idiopathic pulmonary fibrosis.

The expression of 165 (83 upregulated and 82 downregulated) miRNAs in the GSE21394 database was > 2-fold higher in IPF, than in control tissues (Figure 7A and B).

### Construction of miRNA-mRNA network

We integrated target, DEG, and DEM data using Perl to determine interactions between DEMs and DEGs. We identified 13 miRNAs (miR-107, miR-125a-5p, miR-133b, miR-140-5p, miR-142-3p, miR-199a-3p, miR-199a-5p, miR-199b-5p, miR-28-5p, miR-324-5p, miR-34c-5p, miR-429, and miR-520b) and 13 mRNAs (WNT3A, CDH5, DAPK2, EMP2, GPM6A, STXBP6, VEGFA, LRRC32, ADRB1, SLC6A4, CCDC85A, FOXF1, and CXCL14). All miRNAs were upregulated except miR-520b. All genes for these mRNAs were downregulated except CXCL14. We evaluated the interaction information using Cytoscape to create a miRNA-mRNA network (Figure 7C).

## Discussion

Idiopathic pulmonary fibrosis is a chronic and progressive fibrotic pulmonary disease with a poor prognosis 36. Although many factors such as viral infections may trigger or exacerbate IPF, the primary cause remains unknown 37. The incidence of IPF increases significantly with age and the prognosis is poor, as the median survival is 3 years, which is shorter than that for some cancers 37. The pathological features and molecular mechanisms of IPF must be understood to diagnose and effectively treat IPF. The main pathological features of IPF are increased fibroblast proliferation, activation, and aggregation, and collagen synthesis, as well as increased extracellular matrix protein and glycoprotein deposition 38, 39. Some molecules and pathways have been associated with the occurrence and development of IPF, whereas others remain unknown. Microarray and bioinformatic analyses can facilitate better understanding of the pathogenesis of diseases and exploration of biomarkers. We analyzed the GSE10667, GSE15197, GSE47460, and GSE110147 datasets in 86 IPF and 54 normal tissue samples. Because IPF is associated with microRNA, we analyzed miRNA datasets and constructed a miRNA-mRNA regulatory network to explore unknown molecular mechanisms of IPF. We analyzed the GSE21394 miRNA dataset in nine IPF and five normal samples.

We identified 203 DEGs, of which 92 and 111 mRNAs were respectively upregulated and downregulated. The findings of enrichment analyses showed that these genes were mainly involved in immune response (GO: antimicrobial humoral response, antimicrobial humoral immune response mediated by antimicrobial peptide, cell killing, organ or tissue specific immune response; GSVA: immunoglobulin complex, antigen processing and presentation of peptide antigen via MHC class Ib, regulation of T-helper 2 cell cytokine production, positive regulation of natural killer cell mediated immunity). Studies have shown that the pathogenesis of IPF may be related to humoral response 40. In the results of GO and KEGG analyses, we found that IPF may be related to antimicrobial humoral immune response mediated by antimicrobial peptide, which has not been reported. In addition, we also conducted GSVA for each mRNA to uncover DEG biological functions. Among the functions and pathways we have obtained, some functions or pathways have been proved to be involved in the regulation of pulmonary fibrosis, and some have not been reported. Inosine monophosphate (IMP) and uridine triphosphate (UTP) have not been found in IPF, but IMP is involved in the fibrotic process of lupus nephritis (LN) 41. Whether the metabolic processes of IMP and UTP are involved in the process of pulmonary fibrosis awaits further investigation. The functions and pathways associated with the DEGs that we identified through enrichment analysis were also closely associated with IPF. Therefore, we determined biomarkers that are closely associated with the occurrence and development of IPF based on the differential genes identified herein.

Twenty hub genes in IPF had the highest scores in the protein-protein network. Based on these results, we speculated that these predicted genes influence IPF via these enriched pathways and functions. Moreover, we created a miRNA-mRNA regulatory network in IPF based on our findings of 165 differentially expressed miRNAs and mRNAs, predicted miRNA target genes, and DEGs. Our miRNA-mRNA regulatory network included the VEGFA, CDH5, and WNT3A genes that were also hub genes in our PPI analysis. Among them, VEGFA activates NK cells 42. Studies have shown that natural killer (NK) cells can inhibit liver 43 and lung fibrosis 44. We found that VEGFA is downregulated in patients with IPF, which may result in the inhibition of NK cell-mediated immunity, and the subsequent promotion if IPF development. Since WNT3A can also activate NK cells 45, downregulated WNT3A may also contribute to the incidence of IPF. The significance of CDH5 in the pathogenesis of IPF is still unknown. Previous studies have found that Th2 responses damage tissues and fibrotic responses, while Th1 responses ameliorate the latter 46, 47. The function of TH2 cells is negatively regulated by CDH5 48, and downregulated CDH5 may weaken Th2 cell regulation, thus promoting the occurrence and development of IPF.

MicroRNAs are short (19 to 25 nucleotides) single-stranded ribonucleic acids that regulate gene expression after transcription 49. Several past studies showed that the expression of different miRNAs in samples of IPF patients is different from that in control samples 49. Previous studies have found that microRNAs play important roles in occurrence and development of IPF, such as let-7d, miR-154, miR-21, et al. According to Pandit et al., Let-7 can inhibit the occurrence and development of IPF by inhibiting the phenotypic changes of alveolar epithelium 50. The study of Milosevic J, et al. showed that miR-154 was upregulated in IPF and could regulated fibroblast migration and proliferation 51. MiR-21 has also been shown to promote TGFβ1-induced fibrogenic activation of pulmonary fibroblasts in IPF 52. In addition to studies of single miRNAs, miRNAs-mRNAs constitute networks and are involved in many important cellular pathways, which is very important for us to study IPF 53. Previous studies have constructed miRNAs-mRNAs networks using multiple microarray datasets, and found other miRNAs related to IPF through data analysis 54, 55, 56. We also found some miRNAs that may be related to IPF by screening different datasets, and constructed a new miRNAs-mRNAs network. In our study, the miRNAs regulating VEGFA in the network were miR-199b-5p, miR-140-5p, miR-199a-5p, the miRNA regulating CDH5 was miR-125a-5p, and that regulating WNT3A was miR-107. The miRNA, miR-199a-5p, is selectively upregulated in myofibroblasts of the injured lungs in fibroblastic foci of patients with IPF 57. Furthermore, miR-125a-5P is elevated in macrophages exposed to silica, exosomes, recipient fibroblasts, and silicosis serum, suggesting that miR-125a-5p is associated with fibrosis 58. The miRNA, miR-107 is overexpressed in cystic fibrosis 59. The direct target of miR-199a-5P is VEGFA 60, and miR-107 inhibits WNT3A 61, 62. Here, we confirmed that some MiRNAs, genes, pathways, and their regulatory relationships are involved in the development of IPF. However, the mechanisms of some miRNAs and genes in IPF have not been confirmed. The present study uncovered some novel potential biomarkers and molecular mechanisms associated with IPF. We plan to explore whether these biomarkers and mechanisms are involved in the occurrence and development of IPF and if so, to provide novel insights into the diagnosis and treatment of IPF.

We comprehensively analyzed potential genes related to IPF and miRNAs in one miRNA and four mRNA datasets of IPF. Although potential targets for IPF progression have been predicted using microarray analyses 63, 64, the findings of our study are of greater significance. We obtained each GEO dataset using different correction methods. Thus, whether the biomarker results predicted by the matrix file based on background correction are reliable remains unclear. Most microarray analyses are conducted using GEO2R (http://www.ncbi.nlm.nih.gov/geo/geo2r/), express matrix, or single-chip datasets. Here, we used the raw data from Affymetrix (GSE110147) and Agilent (GSE10667, GSE15197, GSE47460) chips. For background correction, standardization and normalization of datasets with the same platform, we used same method to avoid high false-positive rates caused by analyzing individual microarrays and errors caused by the various correction methods used in different datasets. Although the dataset with different platform was processed in different method, it has been proved that it is feasible to integrate the different genes by using RRA package 65. Our approach sheds light on the molecular mechanisms involved in the pathogenesis and treatment of IPF. However, our study has some limitations. We included only one miRNA dataset (GSE21394), with nine IPF and five control lung tissues. The GSE32538 and GSE27430 have miRNA datasets with sufficient samples that meet the requirements 64, but they have been included in other studies. The differences among miRNAs obtained herein were minimal, and further analysis was difficult, which did not meet our inclusion criteria. Therefore, we analyzed GSE21394. Whether the many potential biomarkers and pathways identified herein are actually associated with IPF remains to be determined, and the roles of these molecular mechanisms in the occurrence and development of IPF await clarification.

## Conclusions

We integrated four gene expression datasets to investigate DEMs associated with IPF progression. A total of 203 DEGs and 20 hub genes were identified, which may provide new potential targets for the diagnosis and treatment of IPF. We discovered potentially crucial roles of several functions and pathways in IPF. Besides, three genes identified in our miRNA-mRNA network overlapped with hub genes in IPF. Interactions among miRNAs, mRNAs, and pathways that contribute to the regulation of IPF warrant further investigation.

## Supplementary Material

Supplementary table S1.

### Funding

The fund for the Natural Science Foundation of China (No.81970051), Excellent Top Talent Cultivation Project of Anhui Higher Education Institutions (gxyqZD2017030), the fund from Reserve candidate for Anhui Province Academic and technical leader, and scientific research fund from Anhui medical university (2020xkj257) supported this research.

## Abbreviations

DEG: differentially expressed genes
DEM: differentially expressed miRNA
GO: gene ontology
GSVA: gene set variation analysis
IPF: idiopathic pulmonary fibrosis
KEGG: Kyoto Encyclopedia of Genes and genomes
mRNA: messenger ribonucleic acid
miRNA: micro ribonucleic acid
