# Supplementary material for: Integrated analysis of the molecular mechanisms in idiopathic pulmonary fibrosis
Source: Int J Med Sci. 2021 Aug 2;18(15):3412–24. doi: 10.7150/ijms.61309 (PMC8436110; doi:10.7150/ijms.61309)
Supplement: Supplementary file 1 — Supplementary table S1. [file ijmsv18p3412s1.pdf]

Table S1. 203 integrated DEGs identified by the RRA method.

Name	Pvalue	adjPvalue	logFC
KRT5	5.18E-13	1.30E-08	3.334316256
SPP1	4.68E-12	1.17E-07	3.661034965
MMP1	8.19E-12	2.05E-07	3.924344785
BPIFB1	6.55E-11	1.64E-06	2.940605982
MMP7	1.94E-10	4.86E-06	3.07289576
SAA1	1.02E-09	2.56E-05	2.617139011
ARMC3	1.02E-09	2.56E-05	2.269738524
CP	1.45E-09	3.61E-05	2.758677127
C6	1.67E-09	4.17E-05	2.195239233
TMEM45A	3.50E-09	8.76E-05	2.100187221
PROM1	4.05E-09	0.000101197	2.225330222
KRT15	7.02E-09	0.000175504	2.795022081
SLC27A2	1.17E-08	0.00029347	1.791417492
POSTN	2.07E-08	0.000517173	2.257341476
LTF	2.62E-08	0.000656179	2.014123067
SPAG6	2.87E-08	0.000716479	1.831868685
NEK10	2.97E-08	0.000741727	1.753732284
SPAG17	3.57E-08	0.000892724	1.796397938
EFCAB1	3.81E-08	0.000952971	1.831002628

ZBBX	3.94E-08	0.000984214	1.886807043
COL1A1	4.26E-08	0.00106568	2.510028983
MSMB	6.48E-08	0.001619155	2.023246217
S100A2	9.32E-08	0.002329837	2.615997584
RGS22	9.46E-08	0.002364497	1.695244777
SULF1	9.70E-08	0.00242611	1.679568835
C10orf81	9.95E-08	0.002488562	1.959533154
COL3A1	1.06E-07	0.002651275	2.284923031
IL13RA2	1.14E-07	0.002856443	2.22171354
GPR87	1.19E-07	0.002963381	1.690061921
GSTA5	1.20E-07	0.003007627	1.870088398
LRRIQ1	1.26E-07	0.003148249	1.78171828
CXCL13	1.32E-07	0.003302263	1.643472555
COL14A1	1.42E-07	0.003543795	1.821104374
CHST6	1.52E-07	0.003805496	1.901673417
STK33	1.54E-07	0.003842061	1.688098363
KRT17	1.70E-07	0.004252512	2.244639104
CHST9	1.78E-07	0.004445479	1.583804589
CASC1	1.86E-07	0.004644686	1.665233931
COMP	1.99E-07	0.004986575	2.809244178
C20orf85	2.10E-07	0.005248816	2.017281695

TSPAN1	2.32E-07	0.005800544	1.873700103
CCDC146	2.39E-07	0.005984104	1.586754195
C6orf118	2.49E-07	0.006232761	1.978938023
ABCA13	2.52E-07	0.006296118	1.626343886
TNS4	2.68E-07	0.006698537	2.09202506
SIX1	2.68E-07	0.006698537	1.804319679
FANK1	2.70E-07	0.006753241	1.622278831
LGALS7	2.81E-07	0.007017252	1.886744682
AGR2	2.89E-07	0.007234816	1.49017822
IGF1	3.01E-07	0.007521309	1.637179713
TMPRSS4	3.37E-07	0.008431998	2.588291168
PIP	3.66E-07	0.009143736	1.94751119
SOX2	3.66E-07	0.009143736	1.899294226
CYP24A1	4.20E-07	0.01049886	1.475402236
WDR78	4.59E-07	0.011463663	1.481549634
BPIFA1	4.66E-07	0.011661179	2.202721465
GSTA2	4.85E-07	0.012121072	1.728444406
WDR63	4.87E-07	0.012177541	1.633135982
AK7	5.48E-07	0.01370479	1.465236548
GSTA1	5.84E-07	0.014602466	1.85835725
MNS1	6.15E-07	0.015371381	1.374303235

SPATA17	6.56E-07	0.016389436	1.284019738
RSPH1	6.96E-07	0.017401017	2.181497225
SERPINB3	7.32E-07	0.018291708	1.996907826
RPGRIP1L	7.37E-07	0.018433641	1.315096822
FAM183A	7.70E-07	0.019240289	1.803087901
MMP11	7.95E-07	0.019880982	1.809650585
WDR16	8.21E-07	0.020535729	1.674799975
SPATA18	8.58E-07	0.021449698	1.284334517
MS4A8B	9.03E-07	0.022585816	1.676566955
RHOV	9.03E-07	0.022585816	1.568333646
C10orf107	9.44E-07	0.023599265	1.316403471
CXCL14	9.61E-07	0.024025597	2.439520281
CAPSL	9.91E-07	0.024767859	2.021786547
SFRP4	1.01E-06	0.025179331	1.197097383
SERPINB4	1.08E-06	0.027085958	1.702627229
C11orf88	1.12E-06	0.0278896	1.905801784
TMEM190	1.22E-06	0.030395479	2.022900204
LCN2	1.29E-06	0.032146709	2.05572187
CFH	1.33E-06	0.033181582	1.606721677
LGALS7B	1.43E-06	0.035848173	1.52676559
DSP	1.45E-06	0.036152137	1.30102551

TSGA10	1.46E-06	0.036388618	1.454016773
DIO2	1.47E-06	0.036865059	1.38966459
CRLF1	1.55E-06	0.038803175	2.339738172
SERPINI2	1.57E-06	0.039317819	1.316757171
FCRL5	1.63E-06	0.040847022	1.633216799
KRT6A	1.68E-06	0.041916068	2.083902065
KLHL13	1.91E-06	0.047703864	1.08891493
ASPN	1.93E-06	0.048287391	1.517505311
LOC728763	1.95E-06	0.048631886	1.546037923
WFDC2	1.99E-06	0.049816478	1.348941629
SLC6A4	3.93E-13	9.84E-09	-3.030433224
FCN3	1.33E-12	3.34E-08	-3.024984601
PLA2G1B	1.64E-12	4.10E-08	-2.538787926
AGER	2.87E-12	7.16E-08	-3.169180923
TMEM100	6.30E-12	1.57E-07	-2.52605539
ITLN2	8.19E-12	2.05E-07	-3.238445033
CA4	2.76E-11	6.91E-07	-2.823088885
RTKN2	1.24E-10	3.10E-06	-2.518789223
VIPR1	2.06E-10	5.16E-06	-2.44711642
PEBP4	2.60E-10	6.51E-06	-2.13935559
CACNA2D2	4.41E-10	1.10E-05	-1.891838979

CPB2	5.87E-10	1.47E-05	-2.179657097
CRTAC1	7.66E-10	1.92E-05	-2.216108037
FIGF	1.15E-09	2.88E-05	-2.171665247
SOSTDC1	2.25E-09	5.62E-05	-2.324696043
GPM6A	2.84E-09	7.09E-05	-1.850881081
ABCA3	2.93E-09	7.31E-05	-1.719145955
CLDN18	3.50E-09	8.76E-05	-1.894180043
S100A3	5.47E-09	0.000136668	-1.77524046
PLLP	5.61E-09	0.0001403	-1.847153827
NDRG4	8.35E-09	0.000208852	-1.833583856
LRRC32	1.01E-08	0.000251296	-1.494656525
SPOCK2	1.17E-08	0.00029347	-1.671463047
ST6GALNAC5	1.17E-08	0.00029347	-1.548910315
STX11	1.45E-08	0.000362659	-1.605928203
ACVRL1	1.45E-08	0.000362659	-1.486433333
CAV1	1.81E-08	0.000452176	-1.673330365
NXF3	1.84E-08	0.000461064	-1.950048685
SUSD2	1.95E-08	0.000488514	-1.599777346
DEFA3	2.01E-08	0.000503517	-1.685972541
TNNC1	2.23E-08	0.000557323	-1.911044072
EDN1	2.23E-08	0.000557323	-1.729231656

EMP2	2.31E-08	0.000578253	-1.690375083
SH2D3C	2.87E-08	0.000716479	-1.364386388
BTNL9	4.07E-08	0.001016218	-1.9326139
ESAM	4.20E-08	0.001048997	-1.367458175
C11orf9	4.39E-08	0.001096535	-1.777195987
FGFBP2	4.77E-08	0.001193199	-1.850362343
C1orf198	4.83E-08	0.001206416	-1.484263464
GNLY	6.30E-08	0.001573708	-1.631308121
SMAD6	6.66E-08	0.00166558	-1.456335899
CDH5	6.76E-08	0.001689163	-1.432083753
CA2	6.85E-08	0.001712995	-1.405533069
DAPK2	8.08E-08	0.002019097	-1.551564558
SLCO2A1	9.10E-08	0.002274289	-1.303652284
LGI3	1.05E-07	0.002618186	-1.172546224
GGTLC1	1.06E-07	0.002651275	-1.447751074
SFTA2	1.16E-07	0.002891762	-1.421118823
RGS9	1.17E-07	0.002927407	-1.373601874
CDH13	1.19E-07	0.002963381	-1.429293293
HYAL1	1.20E-07	0.002999685	-1.308453348
HPCAL1	1.31E-07	0.003263241	-1.36002697
HPGD	1.36E-07	0.003390941	-1.599496071

FAM107A	1.38E-07	0.003461861	-1.699832904
HSD17B6	1.55E-07	0.003886159	-1.935979809
S100A12	1.94E-07	0.004850514	-1.66227175
ROBO4	1.96E-07	0.004903022	-1.326503963
TMEM130	2.05E-07	0.00511733	-1.328606955
CLIC5	2.10E-07	0.005248816	-1.70023748
SLC39A8	2.21E-07	0.005520088	-1.695610112
HIGD1B	2.25E-07	0.005625162	-1.479330866
LAMC3	2.32E-07	0.005800544	-1.630148682
VEGFA	2.35E-07	0.005862603	-1.336027297
CAMP	2.44E-07	0.006090335	-1.316008302
SPRY4	2.44E-07	0.006107483	-1.309580189
CSF3	2.68E-07	0.006698537	-1.675631627
ANXA3	3.81E-07	0.009535272	-1.584218509
CYP3A7	3.98E-07	0.009937819	-1.531188681
HBEGF	4.24E-07	0.010592495	-1.381377502
IL6	4.31E-07	0.010776561	-1.880386922
ICAM1	4.47E-07	0.011167524	-1.175165537
DNASE1L3	4.71E-07	0.011765653	-1.122073405
ZBED2	4.85E-07	0.012121072	-1.739924143
CCDC85A	4.85E-07	0.012121072	-1.39653053

HTR3C	5.04E-07	0.012592891	-1.704810724
SHISA3	5.17E-07	0.012924253	-1.3507258
PCDH12	6.05E-07	0.015136195	-1.502551985
ANGPTL4	6.15E-07	0.015371381	-1.305377084
LEPREL1	6.73E-07	0.016815022	-1.277704729
LRRN3	7.20E-07	0.018010302	-1.015742031
FOXF1	7.49E-07	0.018719983	-1.354875264
KL	7.84E-07	0.019599052	-1.123254501
ADRB1	7.90E-07	0.019748519	-1.275833036
ANKRD29	8.71E-07	0.021770436	-1.333433996
IFI27	8.71E-07	0.021770436	-1.340317856
ARC	8.76E-07	0.021887994	-1.462089312
EDNRB	9.03E-07	0.022585816	-1.360389513
SEC14L4	9.10E-07	0.022754257	-1.165271444
GKN2	9.24E-07	0.023089489	-1.358076251
IL1RL1	9.61E-07	0.024025597	-1.306170473
NMUR1	9.79E-07	0.024467593	-1.44729858
AGBL1	9.91E-07	0.024767859	-1.932738992
RP11-165H20.1	9.91E-07	0.024767859	-1.291253372
IL32	1.07E-06	0.026649277	-1.135324606
STXBP6	1.07E-06	0.026837444	-1.373975881

NECAB1	1.07E-06	0.026837444	-1.139500086
ANKRD1	1.09E-06	0.027216766	-1.106158085
DLL4	1.17E-06	0.029174042	-1.26353449
RND1	1.22E-06	0.030605634	-1.307142131
AGTR2	1.34E-06	0.033403346	-1.297566824
APOBEC3A	1.38E-06	0.034528887	-1.360824423
PRX	1.40E-06	0.034897553	-1.236816323
PPP1R15A	1.41E-06	0.035217721	-1.033660707
F11	1.47E-06	0.036626258	-1.510522838
WNT3A	1.55E-06	0.038803175	-1.613854093
RAMP3	1.62E-06	0.040589109	-1.327697429
MME	1.85E-06	0.04631927	-1.184443089
LRRC36	1.86E-06	0.046552693	-1.377794915
HMGCS2	1.87E-06	0.04683851	-1.094848572
KCNMB4	1.92E-06	0.047994962	-1.177196004
GRIA1	1.99E-06	0.049769629	-1.123019002

Table S2. 3 differential KEGG functions and pathways by the RRA method.

Name	Pvalue	adjPvalue	logFC
KEGG_GRAFT_VERSUS_HOST_DISEASE	5.58E-08	1.03E-05	-0.555014209
KEGG_ALLOGRAFT_REJECTION	8.38E-06	0.001541696	-0.470560371

KEGG_TYPE_I_DIABETES_MELLITUS	2.29E-05	0.004212855	-0.40492194

Table S3. 29 differential GO functions and pathways by the RRA method.

Name	Pvalue	adjPvalue	logFC
GO_UTP_METABOLIC_PROCESS	2.44E-07	0.001672155	0.423165069
GO_GTP_BIOSYNTHETIC_PROCESS	6.86E-07	0.004701429	0.405372298
GO_TONIC_SMOOTH_MUSCLE_CONTRACTION	1.48E-06	0.010123888	0.462153741
GO_IMP_METABOLIC_PROCESS	1.95E-06	0.013352293	0.525244974
GO_IMMUNOGLOBULIN_COMPLEX	2.05E-06	0.014009143	0.569067182
GO_EXTRACELLULAR_MATRIX_STRUCTURAL_CONSTITUENT_CONFERRING_TENSILE_STRENGTH	2.08E-06	0.014264599	0.495699962
GO_ALDEHYDE_DEHYDROGENASE_NAD_P_PLUS_ACTIVITY	3.21E-06	0.021978787	0.450113222
GO_OLIGOSACCHARYLTRANSFERASE_COMPLEX	3.67E-06	0.025146931	0.489046848
GO_OLIGOSACCHARYL_TRANSFERASE_ACTIVITY	5.26E-06	0.036052901	0.547721252
GO_EUKARYOTIC_48S_PREINITIATION_COMPLEX	5.73E-06	0.039262836	0.41522642
GO_INTRACILIARY_TRANSPORT_P	6.03E-06	0.041288893	0.519770429

ARTICLE_B			
GO_DYNEIN_LIGHT_CHAIN_BINDING	6.23E-06	0.042682489	0.538165956
GO_NUCLEOBASE_CONTAINING_SMALL_MOLECULE_INTERCONVERSION	6.33E-06	0.04339237	0.399181142
GO_CELLULAR_RESPONSE_TO_ALDEHYDE	6.57E-06	0.044979798	0.525077862
GO_ANTIGEN_PROCESSING_AND_PRESENTATION_OF_PEPTIDE_ANTIGEN_VIA_MHC_CLASS_IB	3.98E-10	2.73E-06	-0.49290883
GO_POSITIVE_REGULATION_OF_VASCULOGENESIS	6.22E-09	4.26E-05	-0.46351363
GO_REGULATION_OF_T_HELPER_2_CELL_CYTOKINE_PRODUCTION	8.59E-08	0.000588453	-0.53066833
GO_POSITIVE_REGULATION_OF_NATURAL_KILLER_CELL_MEDIATED_IMMUNITY	6.86E-07	0.004701429	-0.447832382
GO_MITOGEN_ACTIVATED_PROTEIN_KINASE_KINASE_BINDING	8.95E-07	0.006133853	-0.542996726
GO_REGULATION_OF_INTERFERON_GAMMA_SECRETION	1.21E-06	0.008289945	-0.458369583

GO_T_HELPER_2_CELL_CYTOKINE_PRODUCTION	2.31E-06	0.015846419	-0.429494624
GO_NEGATIVE_REGULATION_OF_NATURAL_KILLER_CELL_MEDIATED_IMMUNITY	2.51E-06	0.017202084	-0.487395367
GO_MHC_PROTEIN_COMPLEX	2.68E-06	0.018365915	-0.450654177
GO_POSITIVE_REGULATION_OF_INTERLEUKIN_5_PRODUCTION	2.94E-06	0.020140684	-0.387928593
GO_T_CELL_TOLERANCE_INDUCTION	2.97E-06	0.020312927	-0.431137134
GO_CD4_POSITIVE_ALPHA_BETA_T_CELL_CYTOKINE_PRODUCTION	3.42E-06	0.023395722	-0.376579976
GO_HEMOGLOBIN_COMPLEX	4.33E-06	0.029686389	-0.489166865
GO_REGULATION_OF_VASCULOGENESIS	4.51E-06	0.030919419	-0.433801651
GO_DENDRITIC_SPINE_HEAD	5.83E-06	0.039901648	-0.399383092
